# Targeted protein degradation in hematologic malignancies: clinical progression towards novel therapeutics

**DOI:** 10.1186/s40364-024-00638-1

**Published:** 2024-08-21

**Authors:** Yupiao Feng, Xinting Hu, Xin Wang

**Affiliations:** 1 Department of Hematology, Shandong Provincial Hospital, Shandong University, No.324, Jingwu Road, Jinan, Shandong 250021 China; 2 Department of Hematology, Shandong Provincial Hospital Affiliated to Shandong First Medical University, No.324, Jingwu Road, Jinan, Shandong 250021 China; 3 Taishan Scholars Program of Shandong Province, Jinan, Shandong 250021 China

**Keywords:** Targeted protein degradation, Hematologic malignancies, Clinical trials, Preclinical, Proteolysis-targeting chimeras, Molecular glue

## Abstract

Targeted therapies, such as small molecule kinase inhibitors, have made significant progress in the treatment of hematologic malignancies by directly modulating protein activity. However, issues such as drug toxicity, drug resistance due to target mutations, and the absence of key active sites limit the therapeutic efficacy of these drugs. Targeted protein degradation (TPD) presents an emergent and rapidly evolving therapeutic approach that selectively targets proteins of interest (POI) based on endogenous degradation processes. With an event-driven pharmacology of action, TPD achieves efficacy with catalytic amounts, avoiding drug-related toxicity. Furthermore, TPD has the unique mode of degrading the entire POI, such that resistance derived from mutations in the targeted protein has less impact on its degradation function. Proteolysis-targeting chimeras (PROTACs) and molecular glue degraders (MGDs) are the most maturely developed TPD techniques. In this review, we focus on both preclinical experiments and clinical trials to provide a comprehensive summary of the safety and clinical effectiveness of PROTACs and MGDs in hematologic malignancies over the past two decades. In addition, we also delineate the challenges and opportunities associated with these burgeoning degradation techniques. TPD, as an approach to the precise degradation of specific proteins, provides an important impetus for its future application in the treatment of patients with hematologic malignancies.

## Introduction

The emergence of molecular targeted therapies, such as small molecule inhibitors, represents a remarkable breakthrough in the tumor therapy. However, approximately 85% of the proteome (such as scaffold proteins and transcription factors), especially some tumor-associated proteins, cannot be effectively targeted by small molecule inhibitors due to the lack of suitable binding sites [1]. In addition, such drugs follow an occupancy-driven pharmacology and require the sustained maintenance of high concentrations of small molecule inhibitors in the treatment regions in order to exhibit their therapeutic effects. This might lead to problems such as accumulation of toxicity, triggering target mutations and development of drug resistance [2–4].

**Fig. 1:**
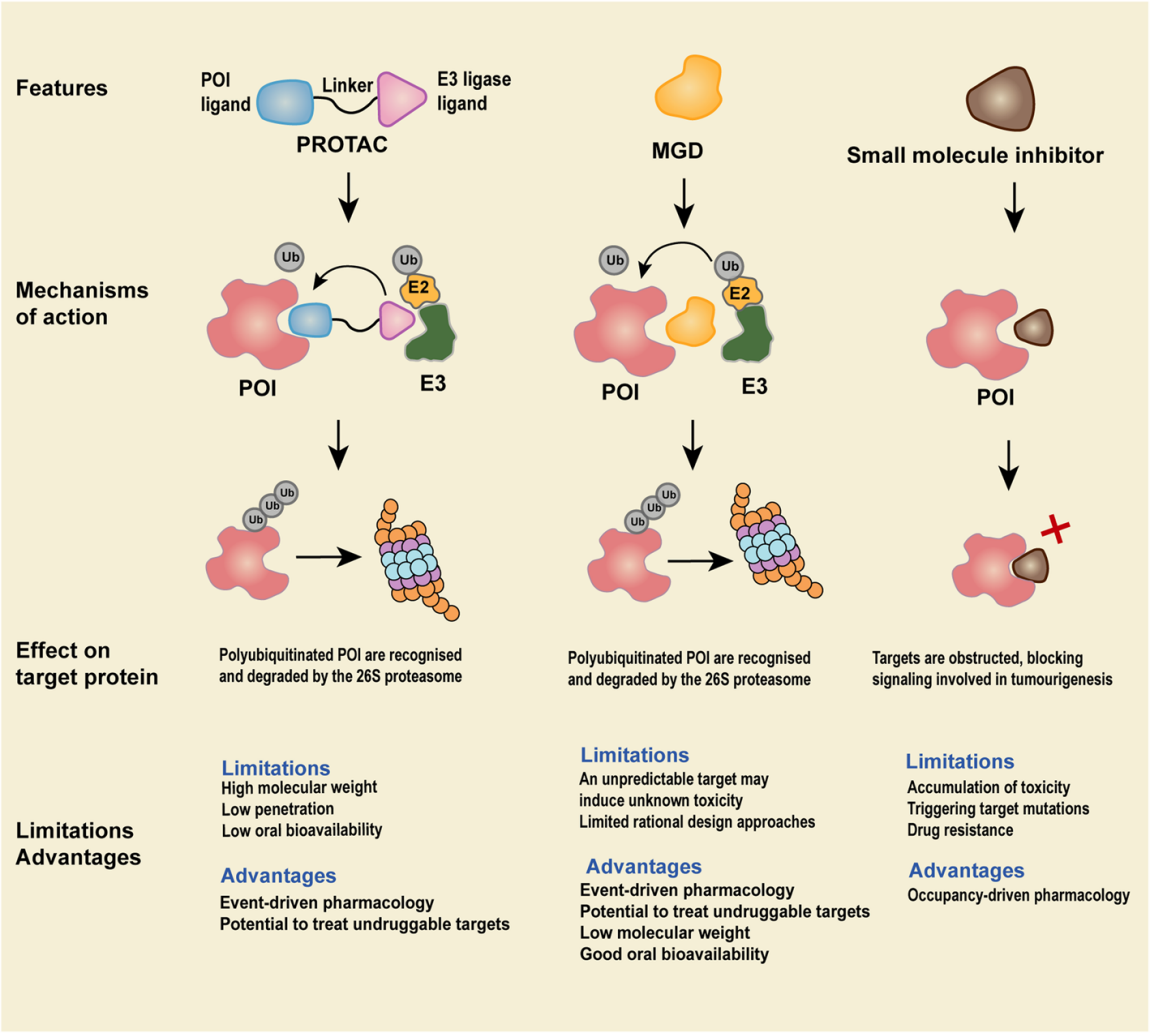
Schematic diagram of the mechanism of action of targeted protein degradation technology and small molecule inhibitors. Targeted protein degradation technologies such as PROTACs and molecular glue, by binding proteins of interest (POI) to E3 ubiquitin ligase, ubiquitination of guided POI. The ubiquitinated POI is then degraded by the 26S proteasome. Small molecule inhibitors work by interacting with specific sites of substrate proteins and further preventing them from participating in downstream signaling pathways related to tumor formation

Targeted protein degradation (TPD) represents as an approach that utilizes small molecule degraders to degrade specific proteins of interest (POI) through endogenous degradation pathways. Harnessing two essential endogenous routes, the ubiquitin–proteasome system (UPS) and the lysosomal pathway, TPD can exert its degradative function [5, 6]. A number of proteins associated with oncogenesis have been found to lack suitable ligand-binding pockets (including intracellular proteins, secreted proteins, and transmembrane proteins), which can be degraded by TPD techniques [7]. Different from conventional drugs that function in an occupancy-driven pharmacology, TPD function in an event-driven pharmacology, allowing for strong degradation of undruggable targets in catalytic amounts **(**Fig. 1**)** [8]. An efficient degradation of POI can be attained through the administration of a minimal dosage of TPD, which concurrently mitigates the occurrence of drug-associated adverse effects. Furthermore, TPD possesses a distinct capability to degrade the entirety of the POI. Consequently, the resistance resulting from mutations in the target protein minimally impacts the degradation functionality of TPD [9, 10].

Introduced in 1999, the concept of TPD was developed to facilitate the specific degradation of POI through the process of ubiquitination [11]. With more than two decades of continuous design and improvement of TPD technologies, various TPD-related degradation techniques have been formed, including but not limited to proteolysis-targeting chimeras (PROTACs), molecular glue, double-mechanism degrader, chaperone-mediated protein degrader, lysosome-targeting chimeras (LYTACs), autophagy-targeting chimeras (AUTACs), autophagosome-tethering compounds (ATTECs) and CMA-based degraders (Table 1). These techniques have significantly broadened the application of TPD and are also providing novel insights into drug discovery. Among these techniques, PROTACs and molecular glue have reached a more advanced stage of development [12]. In addition, since the first molecular glue degrader (MGD), CC-90009, was used in a clinical trial to treat patients with relapsed or refractory acute myeloid leukemia (AML) in 2016, a significant number of targeted protein degraders are now in clinical trials [13]. This implies a swift transition of TPD from a laboratory setting to a clinical setting. Moreover, emerging TPD techniques have shown explosive development in recent years (such as prodrug-based PROTACs (pro-PROTACs), special types of PROTACs, molecular glue, and various TPD techniques based on the lysosomal degradation pathway), which will open up more possibilities for the treatment of tumors [14]. This review describes the action mechanisms of TPD, summarizes relevant data of preclinical experiments (Table 2) and clinical trials on protein degraders (Table 3), particularly PROTACs and MGDs, in patients with hematologic malignancies, and discusses the prospects and potential challenges for the future of TPD.

**Table 1:** Representative targeted protein degradation techniques

Degradation methodology	TPD Technology	Characteristic	Refs
Proteasomal degradation pathway	Proteolysis-Targeting Chimeras (PROTACs)	A heterobifunctional small molecule degrader composed of E3 ligase-binding ligand, POI-binding ligand and linker connecting them	[15]
	Molecular Glue (MG)	Molecular glue reshapes the surface of the CRBN, inducing POI to interact with the E3 ligase	[16]
	Double-mechanism Degrader	A dual-mechanism degrader that chemically induces the degradation of BTK and GSPT1 simultaneously by incorporating PROTACs and MG strategies	[17]
	Chaperone-mediated Protein Degrader (CHAMP)	A heterobifunctional small molecule degrader that is involved in protein folding or recognizes misfolded proteins and guides them through the UPS for degradation	[18]
Lysosomal degradation pathway	Lysosome-Targeting Chimaeras (LYTACs)	A bifunctional molecule with two binding domains, one end of which carries an oligoglycopeptide group that binds to the cell-surface transmembrane receptor CI-M6PR (cation-independent mannose-6-phosphatereceptor), the other end carries an antibody or small molecule that binds to the target protein	[19]
	Antibody-based PROTACs (AbTACs)	A dual antibody that recruits a membrane-bound E3 ligase to degrade cell-surface proteins	[20]
	GlueTAC	GlueTAC was developed for degrading cell-surface proteins and consists of a covalently modified single-domain antibody (nanobody), a cell-penetrating peptide (CPP), and a lysosome-sorting sequence	[21]
	Autophagy-Targeting Chimeras (AUTACs)	The AUTAC consists of three components: a cGMP-based degradation tag, a linker, and a small molecule ligand for a POI or organelle	[22]
	Autophagosome-Tethering Compound (ATTEC)	ATTEC can bind to LC3 and POI, the key proteins of autophagosome, and mediate POI degradation through the lysosomal pathway	[23]
	Autophagy-Targeting Chimeras (ATUOTACs)	AUTOTAC links POI to p62 and leads to POI degradation through the autophagy-lysosomal pathway by promoting the oligomerization and activation of p62	[24]
	CMA-based degrader	CMA-based degrader includes three functional domains: a cell membrane penetration sequence, a POI binding sequence, and a CMA-based targeting motif	[25]

The primary TPD techniques, which stimulate POI degradation via the proteasomal degradation pathway, encompass proteolysis-targeting chimeras (PROTACs), molecular glue, double-mechanism degrader, and chaperone-mediated protein degrader (CHAMP). Conversely, other TPD methodologies instigate POI degradation through lysosomal pathways, incorporating LYTACs, AUTACs, ATTECs, antibody-based PROTACs (AbTACs), GlueTAC, autophagy-targeting chimera (ATUOTAC), and CMA-based degrader

**Table 2:** Preclinical phase of representative targeted protein degraders in hematologic malignancies

Techniques	Degrader	E3	Target	Indications	Refs
PROTACs	RC-1	CRBN	BTK	AML	[26]
PROTACs	PROTACs FLT3	VHL	FLT3	AML	[27]
PROTACs	SD-36	CRBN	STAT3	AML, ALL	[28]
PROTACs	MD-224	CRBN	MDM2	ALL	[29]
PROTACs	PZ15227	CRBN	BCL-XL	ALL	[30]
PROTACs	SIAIS178	VHL	BCR-ABL	CML	[31]
PROTACs	^PMI^Bcr/Abl-R6	MDM2	BCR-ABL	CML/ ALL	[32]
PROTACs	L18I	CRBN, VHL	BTK	DLBCL	[33]
PROTACs	DD-03171	CRBN	BTK	MCL	[34]
PROTACs	MS432	VHL	MEK1/2	MM	[35]
PROTACs	MS943	VHL	MEK1/2	MM	[36]
PROTACs	WL-40	CRBN	Rpn13 homologue	MM	[37]
PROTACs	SS47	CRBN	HPK1	MM	[38]
MGD	CC-885	CRBN	GSPT1	AML	[39]
MGD	GT19715	CRBN	c-Myc / GSPT1	AML/ Lymphoma	[40]
MGD	GT19630	CRBN	c-Myc / GSPT1/2	AML/ Lymphoma/MM	[41]
MGD	SJ6968	CRBN	GSPT1/2	ALL	[42]
MGD	TMX-4116	CRBN	PDE6D, CK1α	MM	[43]
MGD	BI-3802	SIAH1	BCL6	Lymphoma	[44]

*PROTACs *Proteolysis-targeting chimeras, *MGD* Molecular glue degrader, *CRBN* Cereblon, *VHL* Von Hippel-Lindau, *SIAH1* Another type of E3 ubiquitin ligase, *BTK* Bruton’s tyrosine kinase, *FLT-3* The receptor tyrosine kinase FLT-3, *STAT3* Signal transducer and activator of transcription 3, *MDM2* Human murine double minute 2 protein, *BCR-ABL* The oncogenic fusion protein, *MEK1/2* Mitogen-activated protein kinase kinases 1 and 2, *Rpn13 homologue* Proteasome-associated ubiquitin-receptor Rpn13, *HPK1* Hematopoietic progenitor kinase 1, *GSPT1/2* G1 to S phase transition 1 and 2, *CK1α* Casein kinase 1α, *PDE6D* Phosphodiesterase 6D, *AML* Acute myeloid leukemia, *ALL* Acute lymphoblastic leukemia, *CLL* Chronic lymphocytic leukemia, *CML* Chronic myelocytic leukemia, *DLBCL* Diffuse large B-cell lymphoma, *MM* Multiple myeloma

**Table 3:** Clinical trials of representative targeted protein degraders in hematologic malignancies

Techniques	Degrader	E3	Target	Indications	Administration	Phase	Identifier
PROTACs	DT-2216	VHL	BCL-XL	ALL	iv	Phase I	NCT04886622
PROTACs	NX-2127	CRBN	BTK, IKZF1/3	CLL/Lymphoma	Oral	Phase I	NCT04830137
PROTACs	BGB-16673	-	BTK	CLL	Oral	Phase I	NCT05006716
PROTACs	KT-333	**-**	STAT3	Lymphoma	iv	Phase I	NCT05225584
PROTACs	KT-413	CRBN	IRAK4, IKZF1/3	Lymphoma	Oral	Phase I	NCT05233033
PROTACs	NX-5948	CRBN	BTK	Lymphoma	Oral	Phase I	NCT05131022
MGD	CC-90009	CRBN	GSPT1	AML	iv	Phase I	NCT02848001
MGD	E7820	DCAF15	RBM39	AML	Oral	Phase II	NCT05024994
MGD	BTX-1188	CRBN	GSPT1, IKZF1/3	AML	Oral	Phase I	NCT05144334
MGD	CC-122	CRBN	IKZF1/3	Lymphoma	Oral	Phase I /II	NCT01421524
MGD	CC-99282	CRBN	IKZF1/3	Lymphoma	Oral	Phase I /II	NCT03930953
MGD	CC-92480	CRBN	IKZF1/3	MM	Oral	Phase I /II	NCT03374085
MGD	CC-220	CRBN	IKZF1/3	MM/Lymphoma	Oral	Phase III	NCT04975997
MGD	CFT-7455	CRBN	IKZF1/3	MM/Lymphoma	Oral	Phase I /II	NCT04756726
MGD	ICP-490	CRBN	IKZF1/3	MM/Lymphoma	Oral	Phase I /II	NCT05719701

*DCAF15* DDB1 and Cul4-associated factor 15, *IKZF1/3 *Ikaros and Aiolos, *GSPT1 *G1 to S phase transition 1, *RBM39 *A kind of RNA splicing factor, *BTK *Bruton’s tyrosine kinase, *IRAK4 *Interleukin-1 receptor-associated kinase 4, *CDK7/9 *Cycle dependent protein kinase 7/9

## Mechanism of action for TPD

### Protein degradation pathways

**Fig. 2:**
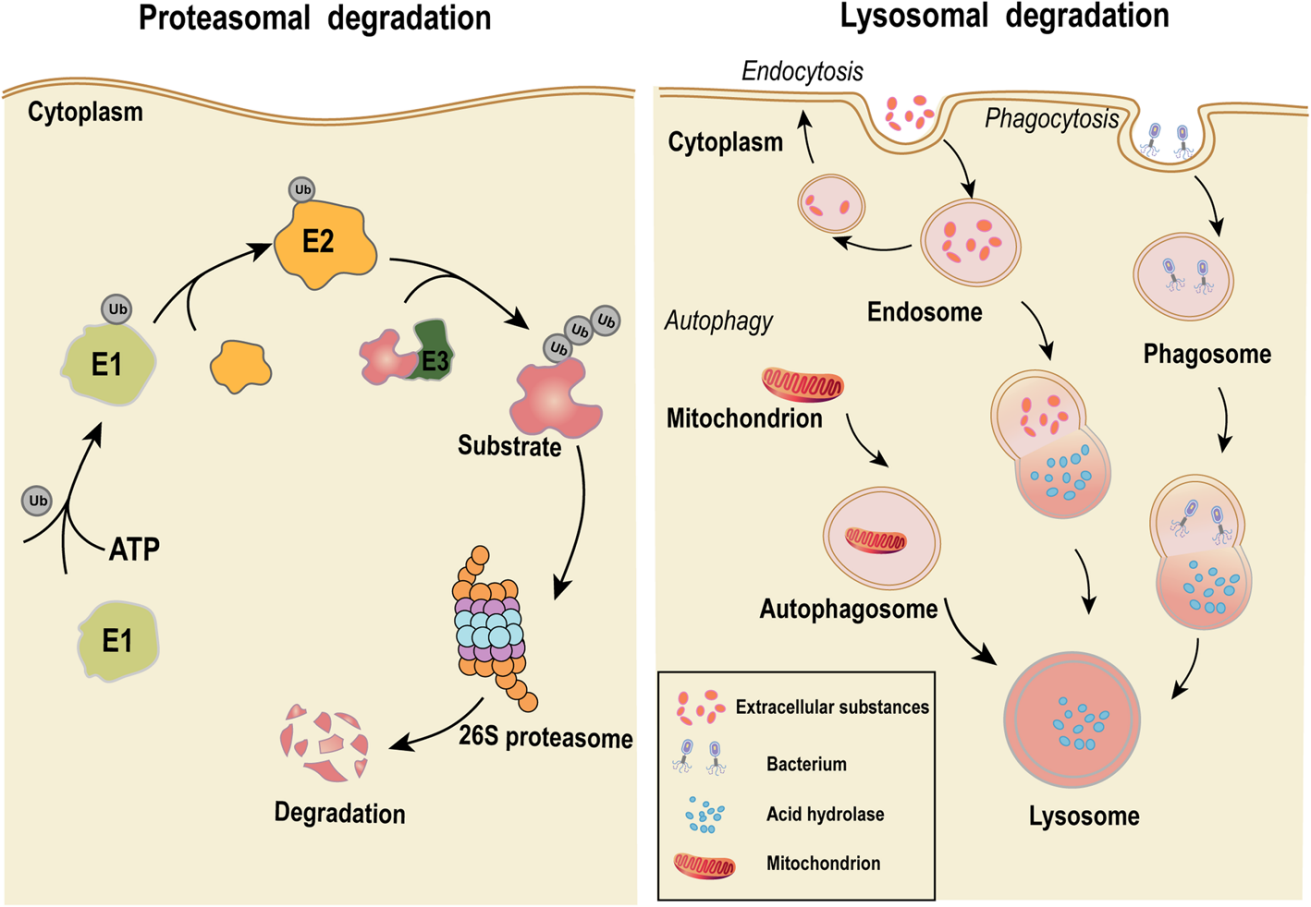
Diagram of the working mechanism of the proteasome pathway versus the lysosomal pathway. In the proteasomal degradation pathway, ATP is involved in E1 ubiquitin-activating enzyme binding to ubiquitin and activating ubiquitin. The activated ubiquitin is transferred to the E2 ubiquitin conjugate enzyme. The E3 ubiquitin ligase transfers ubiquitin to the substrate by binding to the E2 ubiquitin conjugate enzyme. The ubiquitinated substrate will be degraded by the proteasome. In the lysosomal degradation pathway, through endocytosis, phagocytosis, and autophagy, lysosomes can degrade extracellular proteins, large extracellular particles such as pathogens and dead cells, as well as misfolded or aggregated proteins, damaged organelles, and intracellular pathogens

A critical component for achieving the therapeutic impact of the TPD technique is the successful degradation of the pathogenic POI. The UPS and lysosomal degradation pathways represent the principal natural modes of protein degradation in eukaryotic cells, which allow protein degradation in an efficient and specific manner [6]. Intracellular soluble, short-lived, unfolded or misfolded proteins were degraded mainly through UPS by the 26S proteasome [45]. In contrast, dysfunctional organelles (such as degenerated mitochondria), insoluble protein aggregates, and long-lived proteins were degraded mainly by lysosomal pathways [46]. Within the UPS pathway, ubiquitination of POI is achieved by covalently attaching ubiquitin to lysine residues in POI via an enzymatic cascade of E1 ubiquitin-activating enzymes, E2 ubiquitin-coupling enzymes and E3 ubiquitin ligases. Subsequently, the ubiquitinated POI is transported to the 26S proteasome for degradation [47, 48]. Within the lysosomal pathway, substances to be degraded were accepted and processed through endocytosis, phagocytosis, and autophagy processes (Fig. 2) [49]. The primary TPD techniques, which stimulate POI degradation via the UPS, encompass PROTACs, molecular glue, double-mechanism degrader, and chaperone-mediated protein degrader. Conversely, other TPD techniques incorporate LYTACs, AUTACs, ATTECs, and CMA-based degraders, among others, to initiate POI degradation through the lysosomal pathway [50].

### Major E3 ligases in TPD techniques

**Fig. 3:**
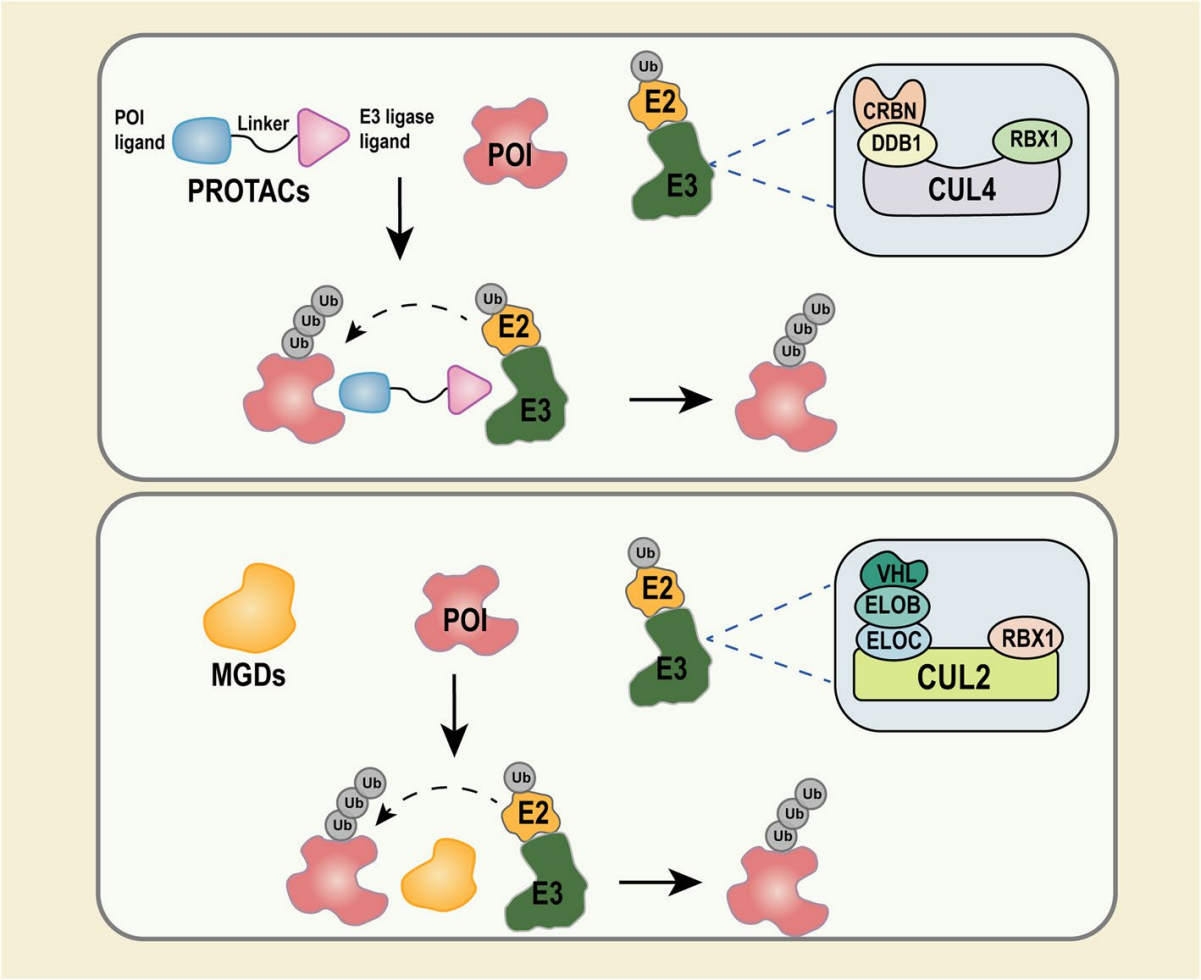
Process of ubiquitination of POI. PROTACs, E3 ligase, and POI form a ternary complex which mediates the ubiquitination of POI. MGDs remodel the surface of E3 ligase and form a ternary complex with E3 ligase and POI, which mediates the ubiquitination of the POI

The E3 ligases serve as an integral element in the action of various TPD techniques. Among the more than 600 E3 ubiquitin ligases identified in human cells, only about 10 are currently involved in the degradation of target proteins, of which Von Hippel-Lindau protein (VHL) and cereblon (CRBN) are two of the more intensively researched [51]. The VHL, Elongin B and C, Cullin 2, and Ring box protein 1 (Rbx1) could form the highly therapeutically promising E3 ubiquitin ligase complex, CUL2-RBX1-EloBC-VHL (CRL2^VHL^) [52, 53]. Initially, VHL proteins were found to cause ubiquitination and degradation of hypoxia-inducible transcription factor (HIFα) by interacting with a subunit of the HIFα [54]. Supported by numerous studies, VHL is now widely applied in TPD techniques and has been successfully used to degrade more than 20 different proteins [55]. CRBN is a substance commonly found in intracellular compartments that interacts with DNA damage binding protein-1 (DDB1), Cullin 4 (Cul4A or Cul4B), and the regulatory factor for Cullins 1 (RoC1) to form the functional E3 ubiquitin ligase complex CUL4-RBX1-DDB1-CRBN (CRL4^CRBN^) (Fig. 3) [56]. The CRBN has been widely applied as an E3 ligase in various TPD techniques, where it can target more than 30 different proteins and has shown its efficacy in various diseases such as cancer, neurodegenerative diseases, and immunological diseases [57]. As the two most widely used E3 ligases in TPD, VHL and CRBN are characterized by the following properties. First, they are extremely flexible and can participate in the degradation of various types of POI in a stable and efficient manner. Second, they are available and relatively accessible. And, finally, their expression is relatively universal and can act on a wide range of diseases. However, there are special cases such as VHL, which has been found to be poorly represented in platelets [58]. Researchers verified this fact by immunoblotting, and VHL E3 ligase expression was almost undetectable in platelets [58].

### Major targets of action in TPD techniques

**Fig. 4:**
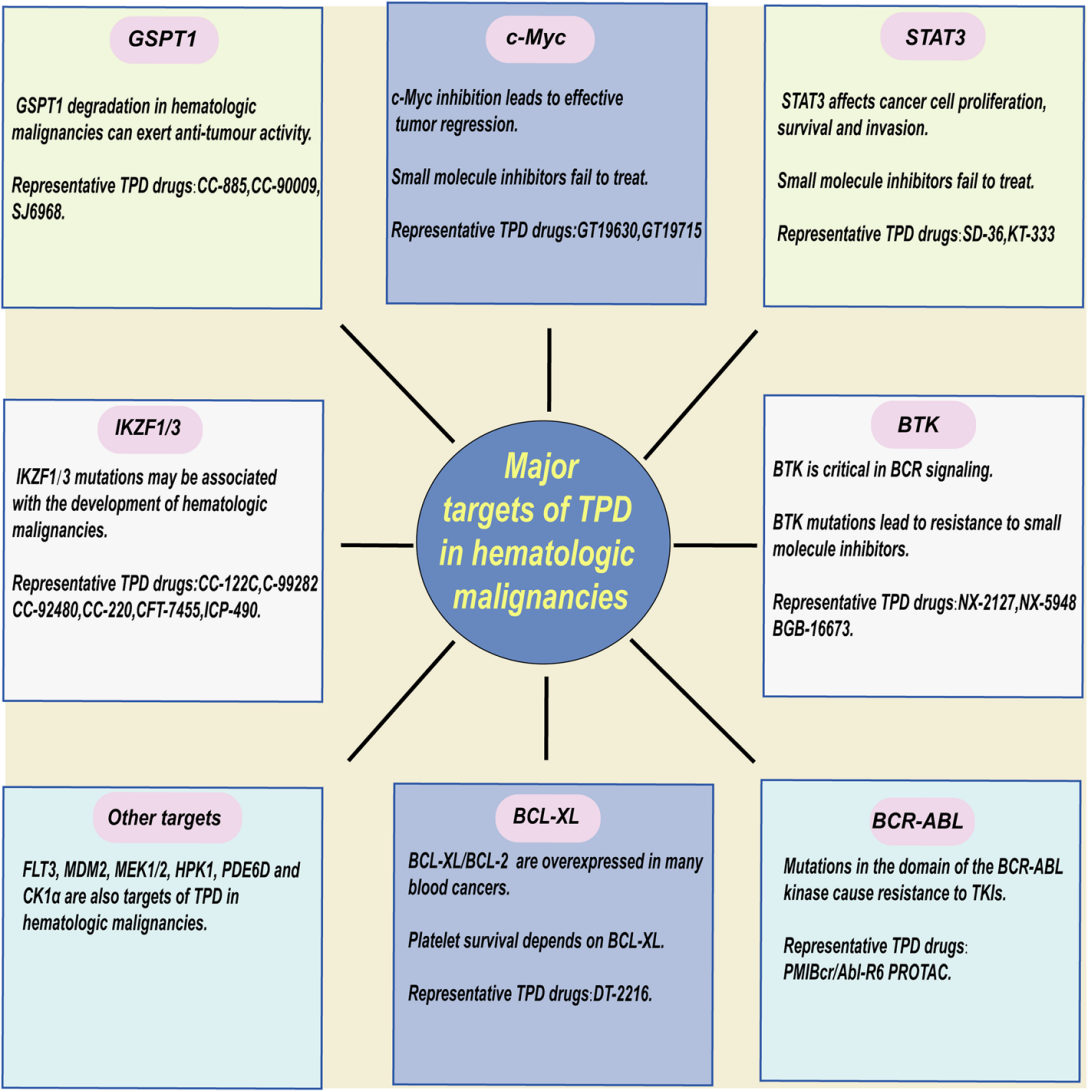
Primary targets for TPD effects in hematologic malignancies. The G1 to S phase transition 1 (GSPT1), Ikaros and Aiolos ((IKZF1 and IKZF3), c-Myc, signal transducer and activator of transcription 3 (STAT3), Bruton’s tyrosine kinase (BTK), BCR-ABL, BCL-XL and other targets serve as the principal targets of TPD action in hematologic malignancies

In the therapeutic field of hematologic malignancies, there are a number of high-value targets associated with the disease that still need to be aimed by effective approaches. For example, undruggable proteins without binding pockets or enzyme activity, target proteins with drug resistance mutations and so on [59]. These primary include Ikaros (IKZF1) and Aiolos (IKZF3), G1 to S phase transition 1 (GSPT1), c-Myc [60], signal transducer and activator of transcription 3 (STAT3) [61], Bruton’s tyrosine kinase (BTK) [62], BCR-ABL [63], and B-cell lymphoma-extra-large (BCL-XL) [64] (Fig. 4). These targets could be targeted by TPD techniques, potentially alleviating the current therapeutic dilemma. Information about these targets is presented in the mechanism of action section for each drug below.

### PROTACs

PROTACs are heterobifunctional small molecule degraders composed of three parts. The part one is a ligand that recruits and binds the E3 ligases, part two is a ligand that binds the POI, and part three is a linker that connects the two ligands. The first PROTAC was identified by the Deshaies’ and Crews’ groups in 2001, which mediated the degradation of methionine aminopeptidase-2 (MetAp-2) by ligating the SCF^β−TrCP^ E3 ligase [11, 65]. Since then, various PROTACs have been developed, the most representative of which was the development of the first small molecule-based PROTAC in 2008 [66]. Targeted degradation could be achieved when POI, PROTACs, and E3 ligase were combined to form the POI-PROTACs-E3 ternary complex [67]. The simple mechanism of how PROTACs degrades POI via UPS is the following: in the presence of ATP, the E1 ubiquitin activator enzyme binds to ubiquitin and activates it, after which the activated ubiquitin is transferred to the E2 ubiquitin conjugate enzyme and the E3 ubiquitin ligase binds to the E2 conjugate enzyme, and finally the POI is ubiquitinated. When the process described above was repeated, POI occurred polyubiquitination, which was degraded in the 26S proteasome. At the same time, PROTACs were recycled [68].

The E3 ligand is a determinant of target selectivity and potency of PROTACs [69]. Some of the most common E3 ligands in PROTACs are VHL ligand, CRBN ligand, mouse double minute 2 homolog protein (MDM2) ligand, and inhibitor of apoptosis proteins (IAPs) ligand. The first VHL ligand was a heptapeptide (ALAPYIP), which was discovered in the background of VHL E3 ligase-mediated degradation of HIF-1α [70]. Subsequently, with the help of computers, Crews’ group discovered small-molecule VHL ligands [71]. Building on this, the researchers optimized its structure to enhance its affinity for the VHL E3 ligase. The most meaningful case in the exploration of CRBN ligands is that thalidomide mediates the degradation of IKZF1/3. The zinc finger transcription factors IKZF1 and IKZF3 are implicated in regulatory mechanisms and multiple signaling pathways, which are crucial for the stability of the internal environment and the development of immune cells. The overexpression of IKZF1/3 is closely associated with the development of several hematologic malignancies [72]. However, it has also been reported that pomalidomide may ubiquitinate and degrade zinc finger proteins, which perform critical biological functions in disease progression and normal development. The off-target degradation of these key proteins may have long-term effects such as dysregulation of lymphocyte development, development of new cancers and teratogenic effects [73]. Therefore, probing suitable CRBN ligands is crucial for TPD techniques. Immunomodulatory drugs (IMiDs) have glutarimides that bind to CRBN and can directly penetrate the hydrophobic pocket within the CRBN E3 ligase [74].

Among POI ligands, it ranges from reversible and irreversible covalent and non-covalent ligands to allosteric ones. They can be directed to more than 100 targets including receptor tyrosine kinases (like FLT3, HER3, EGFR), fusion proteins (like BCR-ABL), chromatin readers (like BRD4), aggregation-prone misfolded proteins (like Tau). Moreover, there are databases that collect information on the chemical structure, physicochemical properties, and biological activity of POI ligands [75].

The linker, as part of the composition of PROTACs, is not an inert bridging structure which can significantly modulate the interaction between POI and E3 ligases. The structures range from simply alkyl and polyethylene glycol (PEG) chains to firmer piperazine/piperidinyl linkers [76]. The bioactive profile of PROTACs can be modified after rational adjustment of the linker properties [77]. It has been found that when the atomic composition, length, and flexibility of the linker are altered, the biological activity properties of PROTACs, such as PKs, cell permeability, potency, and isoform selectivity, are also altered [78, 79]. However, most linker structure–activity relationship researches are experimental and linker design remains a limitation [76].

### Molecular glue degraders

Molecular glues are monovalent molecules that are also capable of inducing the formation of ternary complexes between POI and E3 ligases, and when ubiquitination of POI is complete, the polyubiquitinated POI are transferred to the proteasome for degradation [16, 80]. The original case for molecular glues came from FK506 and cyclosporin A (CsA), which induced the formation of FKBP12-FK506-Calcineurin and cyclophilin-CsA-Calcineurin complexes, respectively, hence the name “molecular glues” [81]. Molecular glues were first introduced into clinical practice due to their structural superiority, usually following the “Lipinski’s rule of five”. Compared to PROTACs, molecular glues have lower relative molecular masses, simpler chemical structures, better cell permeability, and superior drug formation properties. However, the current discovery of molecular glue is mainly serendipitous and lacks rational design strategies, leading to limitations in the exploration efficiency and applicability of molecular glues [80].

## Recent advances of targeted protein degradation in hematologic malignancies

Coincidentally, long before the mechanism of action of TPD was understood, drugs based on TPD techniques were already in clinical application. IMiDs were the first drugs approved by the FDA for hematologic malignancies. In 2010, related studies found that thalidomide could bind to the substrate receptor CRBN of E3 ubiquitin ligase complex 4, which led to a better understanding of the mechanism of action of TPD [82–85]. Cereblon E3 ligase-modulating drugs (CELMoDs) are novel thalidomide derivatives with complex structures that also contain a glutarimide ring as part of their base structure, which binds directly to CRBN with high affinity [86–88]. Nowadays, numerous PROTACs and MGDs are rapidly entering the therapeutic field of oncology, and in the following section we will present as comprehensive a view as possible of the drugs that are currently in preclinical experiments or clinical trials in hematologic malignancies (Table 4).

**Table 4:** Latest efficacy of targeted protein degraders in hematologic malignancies

Techniques	Drug	Phase Identifier	Patient Number	Treatment	Efficacy	G3/4 AEs	Refs
PROTACs	NX-2127	Phase I NCT04830137	28(17CLL)	Monotherapy	ORR (33%) (CLL)	Fatigue (62%) Neutropenia (39%) Anemic (27%)	[89]
PROTACs	BGB-16673	Phase I/ II NCT05006716	26(10CLL)	Monotherapy	1CR(MCL) 5PR(CLL)	Contusions (30.8%) Fever (23.1%) Neutropenia (23.1%)	[90]
PROTACs	KT-333	Phase I NCT05225584	21(13ST, 7NHL, 1HL)	Monotherapy	1PR(CTCL) 3SD(ST)	Anemia Nausea Fatigue	[91]
PROTACs	NX-5948	Phase I NCT05131022	14(4CLL, 10NHL)	Monotherapy	1PR(CLL) 2SD(CLL)	Petechiae/bruising (57.1%) Thrombocytopenia (35.7%) Nausea (35.7%)	[92]
MGD	CC-90009	Phase I NCT02848001	45(AML)	Monotherapy	1CR,1CRi, 1MLFS	Infections (47%) Hypocalcemia (22%) Hypotension (13%)	[13]
MGD	E7820	Phase II NCT05024994	12(7 AML,5 MDS)	Monotherapy	1mCR(MDS)	G3/4 AEs (75%)	[93]
MGD	CC-122	Phase I NCT01421524	97(DLBCL)	Monotherapy	ORR (29%) CR (11%)	Neutropenia (51%) Infection (24%) Anemia (12%)	[94]
MGD	CC-99282	Phase I/ II NCT03930953	35(30 DLBCL, 5FL)	Monotherapy	ORR (40%) 3CR 7PR	Neutropenia (54%) Thrombocytopenia (9%) Febrile neutropenia (6%)	[95]
MGD	CC-92480	Phase I/ II NCT03374085	101(MM)	CC-92480 DEX	ORR (39.6%) 2sCR 3CR 18VGPR 17PR	Neutropenia (74.3%) Anemia (32.7%) Thrombocytopenia (25.7%)	[96]
MGD	CC-220	Phase III NCT04975997	38(MM)	CC-220 DEX	ORR (36.8%) 2CR 5VGPR 7PR	Neutropenia (50%) Anemia (28.9%) Leucopenia (23.7%)	[97]

*DEX* Dexamethasone, *CR* Complete remission, *ORR* Overall response rate, *VGPR* Very good partial remission, *MLFS* Morphologic leukemia free state, *AEs* Adverse events, *ST* Solid tumor

### Acute myeloid leukemia

Acute myeloid leukemia (AML) is a malignant clonal disease of hematopoietic tissue, marked by the aggregation and expansion of immature myeloid cells in the bone marrow and peripheral blood, resulting in the failure of normal hematopoietic functions [98, 99]. AML is a leukemia that predominantly affects adults, where its prevalence rises significantly with the age of the patient [99]. In AML patients, the formidable hurdle of relapsed or refractory therapeutic response still necessitates resolution. The utilization of targeted protein degraders in the treatment of AML presents a promising solution to this challenge. Presently, drugs such as CC-90009, E7820, BTX-1188 and CC-885 are showing initial benefits in AML treatment.

#### CC-885

CC-885 is a CELMoD containing a glutarimide ring that can bind to CRBN E3 ligase like IMiDs. It has anti-tumor effects due to its targeted degradation of GSPT1 in AML [39]. GSPT1, a small GTPase, collaborates with the translation termination factor eRF1 to facilitate the concluding stage of protein biosynthesis, namely, translation termination [100]. When GSPT1 is degraded by CC-885, it leads to wide-spectrum growth inhibition in patient-derived AML and cancer cell lines. This indicates potential clinical efficacy of CC-885 in the treatment of hematologic malignancies [39]. Furthermore, it was also reported in 2020 that CC-885 could target the BCL-2 interacting protein 3 like (BNIP3L) [101]. BNIP3L serves as an integral dual regulator for both programmed cell death pathways and mitochondrial turnover. In the absence of BNIP3L, AML cells demonstrate amplified sensitivity towards mitochondrial-targeted pharmacological agents [102, 103]. When CC-885, E3 ubiquitin ligase, and BNIP3L form the complex, ubiquitinated BNIP3L is degraded in the proteasome, which provides a putative combination of CC-885 with mitochondria-targeted drugs for the treatment of AML [101]. However, the development of CC-885 has been hampered by the discovery that CC-885 degrades a wide range of target proteins, such as GSPT1, BNIP3L, CK1α, HBS1L, IKZF1, and IKZF3, with uncontrolled off-target toxicity [39, 101]. There are no reports of clinical trials for CC-885 currently.

#### CC-90009

Stimulated by the deficiency of CC-885 in degrading a range of target proteins, the selective degradation of GSPT1 has emerged as a latest thinking. CC-90009 is a CELMoD that selectively degrades GSPT1, which can eliminate disease-driven leukemic stem cells thereby exerting anti-AML activity [100]. It is reported that CC-90009 has potent anti-proliferative activity both in AML cell lines and primary cells, but has minor influence on peripheral blood mononuclear cells (PBMCs) from healthy donors [100]. Following an additional validation study, it was observed that treatment with CC-90009 led to rapid and efficient diminishment of surviving leukemia cells from AML patients. However, in a stark contrast, the normal lymphocytes within the same patient displayed a reduced reaction to CC-90009. The investigators evaluated the impact of CC-90009 on the overall proteome in AML cells by mass spectrometry and found that GSPT1 protein abundance was reduced after CC-90009 treatment. Moreover, immunoblotting demonstrated selective degradation of GSPT1, which was not found in target proteins such as CK1α, HBS1L, and IKZF1 [100]. This CELMoD is presently undergoing clinical trials, either as monotherapy (NCT02848001) or in conjunction with gilteritinib, venetoclax, or azacitidine (NCT04336982). These trials aim to investigate its potential efficacy in AML and/or myelodysplastic syndrome (MDS) [100].

CC-90009 selective degradation of GSPT1 could be influenced by a variety of factors. ILF2 and ILF3 are regulators of RNA alternative splicing, and the heterodimeric complexes formed by them act as novel regulators of CRBN expression. Knockdown of ILF2/ILF3 regulated the selective splicing of CRBN messenger RNA and reduced the generation of full-length CRBN protein, leading to an attenuated response to CC-90009 [100]. In addition, deletion of TSC1 or TSC2 leads to hyperactivation of mTORC1, which can hamper the binding of GSPT1 to CRBN induced by CC-90009 [100, 104]. Subsequently, the reduction in the degradation of GSPT1 and the sensitivity of CC-90009 to AML were reduced [100]. The degradation of GSPT1 promotes the activation of critical constituents in integrative stress response pathway ( like ATF4, GCN1, and GCN2), while inducing acute apoptosis in AML cells [100].

The dose-finding study (NCT02848001) for CC-90009 in the treatment of relapsed/ refractory AML is currently ongoing. In 45 patients with relapsed/refractory AML treated with CC-90009, a dose-dependent decrease in GSPT1 levels was observed in T cells and peripheral blood cells, with a greater decrease in levels as the dose increased. The findings identified 1 complete remission (CR), 1 morphologic complete remission with incomplete blood count recovery (CRi), and 1 morphologic leukemia-free state (MLFS). Grade 3/4 treatment-emergent adverse events (TEAEs) associated with CC-90009 were primarily infections (47%), hypocalcemia (22%), and hypotension (13%). Currently, this research is ongoing, with the hope of reducing toxicity by optimizing the dose and duration of therapy [13]. In addition, CC-90009 was also shown to have a synergistic effect when combined with IDH2, BCL-2, and FLT3 inhibitors to further enhance the prognosis of patients with AML [105].

#### E7820

DDB1 and Cul4-associated factor 15 (DCAF15) is one of the less common E3 ligases used in TPD techniques [106]. When DCAF15 ligase complex binds to RNA splicing factor RBM39, E7820 can mediate degradation of RBM39 [107, 108]. Patients with AML and MDS often have mutations in RNA splicing factor genes ( SRSF2, SF3B1, ZRSR2 and U2AF1) [109]. The RBM39 is essential for the survival of AML cells with mutations in the splicing factor genes, and has been associated with a poor prognosis in AML. As an MGD, E7820 can degrade RBM39 in animal models of bone marrow malignancies with splicing factor mutations and in vitro experiments, leading to complete interruption of mRNA splicing [93]. In a phase II clinical trial (NCT05024994) involving 12 patients (7 AML, 5 MDS), E7820 was used primarily to treat patients with splicing factor gene mutations in relapsed or refractory myeloid malignancies. As of 14 July 2022, 1 patient with MDS had a transient major cytogenetic response (mCR), and the condition of all other patients is steady or progressing with optimal response. Of the adverse events treated with E7820, 9 patients (75%) experienced grade 3/4 adverse events and 6 patients (50%) experienced serious adverse events [93]. E7820 monotherapy was found to have tolerable safety outcomes in the phase II trial. The degradation of the RBM39 has shown an example of how splicing factor mutant diseases can also be a therapeutic target.

#### BTX-1188

BTX-1188 is an MGD which targets GSPT1 and IKZF1/3 for dual degradation in the therapy of solid tumors and AML. Within the realm of hematologic malignancies treatments, BTX-1188 demonstrates a number of distinguishing characteristics. One is that it targets GSPT1 and IKZF1 to achieve dual degradation of POI. And BTX-1188’s degradation of GSPT1 is persistent. Then, BTX-1188 degradation of IKZF1/3 demonstrated its immunomodulatory properties, which were manifested by its ability to inhibit pro-inflammatory cytokines (IL1b, IL-6, TNF-a) and to stimulate IL-2 induction in PBMC via aCD3 and LPS. BTX-1188 has the benefit of simultaneous degradation of IKZF1/3, thus avoiding the toxicity related to the degradation of GSPT1 alone. BTX-1188 showed cytotoxicity against a variety of cancer cell lines (such as MYC-dependent, non-MYC-dependent cell lines, and primary human AML patient samples). These unique properties make BTX-1188 a promising drug candidate for therapy in AML [110]. Currently, BTX-1188 has entered clinical trials in patients with advanced malignancies (NCT05144334).

### Acute lymphoblastic leukemia

Acute lymphoblastic leukemia (ALL), a hematologic malignancy originating from immature B and T lymphocytes, presents contrasting prognosis across different age groups. In pediatric patients, the prognosis is relatively favorable with a cure rate exceeding 90%. Conversely, the success rate for ALL cure in adults is significantly lower, standing at less than 40% [111, 112]. Methotrexate is the fundamental drug in the treatment of ALL, but its efficacy is not satisfactory due to drug resistance [113]. Even after extensive chemotherapy and hematopoietic stem cell transplantation (HSCT), patients with relapsed or refractory ALL still have a poor clinical prognosis [114]. TPD drugs such as SJ6986 and DT-2216 have also been developed in recent years for the treatment of ALL.

#### SJ6986

SJ6986 is a CELMoD that selectively targets degradation of GSPT1/2, which has been shown to be effective in patients with ALL. SJ6986 has been proven to have anti-ALL properties in xenograft mouse models and multiple ALL cell lines. In addition, SJ6986 has shown significant effects in the single drug treatment of high-risk ALL patient-derived xenograft (PDX) models, including the treatment of hypodiploid ALL, Ph-like ALL, and ZNF384 rearranged mixed-phenotype acute leukemia. The mechanism of action of SJ6986 is to induce cell apoptosis by inhibiting the transition from S to G2/M phase of the cell cycle. It is worth noting that SJ6986 has minimal impact on normal hematopoiesis [100]. No clinical trials have been documented for SJ6986, which is still in preclinical studies.

#### DT-2216

The overexpression of anti-apoptotic proteins (including BCL-2, BCL-XL, and MCL-1) has been associated with the ability of tumors to evade apoptosis. BCL-XL, in particular, has been identified as being overexpressed in a myriad of both leukemia and solid tumor cells. Moreover, this overexpression is associated with increased resistance to cancer drugs [115, 116]. Related studies have found that AML progression from JAK2-mutant (JAK2-mut) myeloproliferative neoplasms (MPN) (after MPN AML) has a poor prognosis, and the BCL-XL is overexpressed in the cells of patients with JAK2-mut MPN [117]. Consequently, targeting the anti-apoptotic proteins have emerged as a viable approach for cancer treatment. An example worthy of attention is ABT263 (navitoclax), a dual inhibitor of BCL-XL and BCL-2. However, given that the inhibition of BCL-XL by ABT263 leads to thrombocytopenia as a side effect, we urgently need to develop a more efficient and less side-effect BCL-XL targeted drug [118–120].

Based on PROTACs technology, DT-2216 is a selective BCL-XL degrader. The E3 ligase connected to DT-2216 is VHL, which has relatively low expression in platelets, so the toxic effect on platelets is minimal when DT-2216 exerts its anti-tumor effect. There is evidence that DT-2216 can act in numerous BCL-XL-dependent cancer and leukemia cells [58]. DT-2216 performed well in preclinical models of T-ALL and effectively cleared leukemia cells. In experiments with in vitro T-ALL cell lines, there was a dose-dependent decrease in cell viability of T-ALL lines after DT-2216 treatment. In a living T-ALL mouse xenograft model, DT-2216 reduced the leukemia burden and delayed leukemia progression [121]. Moreover, DT-2216 has also been found to target BCL-XL-dependent T-cell lymphoma cells, selectively killing the cells with no significant platelet toxicity [122]. Study of DT-2216 to treat relapsed or refractory malignancies is recruiting (NCT04886622).

### Chronic lymphocytic leukemia

Chronic lymphocytic leukemia (CLL) is one of the most common adult leukemias and presents as a malignant proliferation of mature monoclonal B lymphocytes in bone marrow, blood and lymphoid tissues [123]. The majority of CLL patients usually receive chemoimmunotherapy, however, elder patients can only receive low-intensity chemoimmunotherapy owing to poorer tolerances [124, 125]. Despite the significant progress in the treatment of CLL through the utilization of molecularly targeted therapeutics, numerous patients inevitably face the undesirable prospect of relapsed or refractory conditions [123, 126].

BTK mutations can lead to impaired conversion of promyeloid B cells to mature peripheral B cells [127, 128]. The BTK forms a critical constituent of the B cell receptor (BCR) signaling pathway [89]. It is noteworthy that excessive activation of the BCR signaling is frequently correlated with disease progression in CLL [129–132]. In mouse models of CLL, studies have demonstrated that a deficiency of BTK results in either the prevention or deferment of tumor development. Conversely, an overexpression of BTK expedites the progression of leukemia, thereby exacerbating mortality rates [133]. Consequently, the strategic degradation of BTK presents potential therapeutic advantages for patients diagnosed with CLL. Covalent BTK inhibitors, such as zanubrutinib, acarbrutinib, and ibrutinib, have demonstrated favorable efficacy during treatment of CLL by inhibiting BTK. However, the development of a BTK-resistant mutation occurring at cysteine 481 poses a challenge to the successful binding of these BTK inhibitors. To tackle this obstacle, non-covalent BTK inhibitors (such as pirtobrutinib) were developed with the expectation of overcoming the BTK C481 mutation. While this approach has shown significant efficacy in CLL patients with this specific mutation, its effectiveness is compromised by the surfacing of novel BTK mutations during the progression of the disease [134]. Consequently, TPD has surfaced as an exceptionally promising therapeutic strategy to combat CLL resistance.

#### NX-2127

NX-2127 is a PROTAC that mediates dual degradation of BTK and IKZF1/3 [135, 136]. In a preliminary study of the efficacy of NX-2127 in patients with B-cell malignancies and relapsed or refractory CLL, a total of 28 patients (including 17 patients with CLL) participated in the clinical trial (NCT04830137). All patients had been administered BTK inhibitors in the past, and a significant proportion-76.5%, had also undergone treatment with venetoclax. As of 16 June 2022, the average BTK degradation rate was 86% in all patients in cycle 1, and 83% in CLL patients. Among the study’s participants, there were 12 patients with CLL whose responses could be evaluated. With a final overall response rate (ORR) of 33% for NX-2127, the evidence suggests that the ORR increases with longer time to follow-up (16.7% at 2 months, 42.9% at 4 months, and 50% at 6 months). In addition, patients with disease progression with non-covalent BTK inhibitors and patients with BCL-2/BTK inhibitors double refractory also responded to NX-2127. The main adverse events that occurred during treatment were fatigue (62%), neutropenia (39%), anemia (27%), hypertension (27%). The provided data implies that NX-2127 has exhibited promising results in cases pertaining to relapsed or refractory CLL and B-cell malignancies [89].

#### BGB-16673

BGB-16673, developed upon the basis of PROTACs techniques, serves as a BTK-dependent degrader. It is ingeniously designed for the systematic degradation of multiple mutant forms, alongside the wild-type BTK [137]. In preclinical studies on TMD-8 lymphoma cells expressed wild-type BTK as well as L528W, C481S and T474I mutants, BGB-16673 demonstrated potent antiproliferative activity, even superior to covalent/non-covalent BTK inhibitors. In a mouse xenograft model carrying the BTK C481S, T474I, and L528W mutations, BGB-16673 resulted in complete tumor regression and demonstrated long-lasting antitumor activity and low metastasis rates [134].

As of 26 May 2023, a total of 26 patients with malignant B-cell malignancies, including 10 patients with CLL, have participated in the clinical trial (NCT05006716). Upon preliminary pharmacodynamics (PD) data analysis, we observed that BTK protein levels in tumor tissue as well as in peripheral blood are significantly reduced even at the lowest dose. Of the 18 patients evaluable for response, 12 (67%) responded, including 1 mantle cell lymphoma (MCL) patient who experienced a CR. Of the 6 CLL patients who responded, 5 achieved PR. The TEAEs were experienced by 88.5% of the patients. The most frequently occurring of these included contusions, recorded in 30.8% of cases, and fever, neutropenia, and elevated lipase levels, each reported in 23.1% of cases [90].

### Lymphoma

Lymphomas, prevalent malignancies within the hematologic system, predominantly comprise non-Hodgkin’s lymphoma, accounting for approximately 80%-85% of cases. Conversely, Hodgkin’s lymphoma is significantly less common, representing a mere 10%-15% of instances. Non-Hodgkin’s lymphoma (NHL) comprises a heterogeneous group of tumors, such as the relatively indolent follicular lymphoma (FL) and the aggressive diffuse large B cell lymphoma (DLBCL) [138]. DLBCL as the most common subtype of NHL has a relapse rate of up to 45%-50% in patients treated with the standard regimen of chemoimmunotherapy with rituximab, cyclophosphamide, doxorubicin, vincristine and prednisone (R-CHOP) [139]. Certain patients with other subtypes of lymphoma also have difficulty avoiding relapsed and refractory conditions. Thus, an array of targeted protein degraders, including CC-122, CC-99282, KT-333, KT-413, NX-5948, is expected to demonstrate significant potential in lymphoma therapy.

#### CC-122 (Avadomide)

CC-122, classified as a CELMoD, is capable of mediating the degradation of IKZF1/3. The CC-122 demonstrates the capacity to inhibit the growth and induces the apoptosis, of DLBCL cell lines and malignant B cells [140]. CC-122 induced IKZF1/3 degradation would cause increased apoptosis in both germinal center B-cells and activated B-cell DLBCL cell lines [141]. In clinical trial (NCT01421524) enrolling 97 patients with relapsed/refractory DLBCL, 3–5 mg of CC-122 was administered to patients. As of April 2018, ORR was 29%, CR was 11%, and the prevalent grade 3/4 TEAEs on the list were neutropenia (51%), infection (24%), anemia (12%) and febrile neutropenia (10%) [94]. While CC-122 treatment demonstrates significant efficacy in most patients, a subset of DLBCL patients continue to exhibit poor responsiveness. The likely cause is that resistance is conferred by pre-existing or acquired genetic or epigenetic lesions in malignant B cells [142]. Moreover, the researchers found that deletion of KCTD5, TRAF2, TRAF3, NFKBIA, RFX7, AMBRA1, or CYLD genes reduced the anti-DLBCL activity of CC-122 by genome-wide CRISPR screen [142].

#### CC-99282 (Golcadomide)

The compound C-99282, colloquially termed golcadomide (GOLCA), is a specific classifier within the CELMoD, engineered to target the degradation of IKZF1/3 [143]. Exhibiting superior efficacy over parallel drugs designed for the same purpose in hematologic malignancies, such as CC-220 and lenalidomide, CC-99282 delivers accelerated, more profound, and longer-lasting IKZF1/3 degradation [144]. Specific information about CC-220 is described in the MM section. The potent degradative effect of CC-99282 on IKZF1/3 results in the derepression of interferon-stimulated genes (such as IRF7) and cell cycle-dependent kinase inhibitors, as well as the reduction of c-Myc, a key oncogene, leading to effective autonomous cell killing and apoptosis in a broad range of genetically heterogeneous NHL cell lines [145]. Moreover, the CC-99282 has excellent distribution advantages, favoring target tissues like lymphoid organs, thus showing enhanced anti-proliferative and apoptotic activities among various lymphoma cells [144].

The efficacy and safety of CC-99282 for relapsed or refractory NHL was primarily evaluated in a clinical study (NCT03930953) that consisted 35 patients, including 30 with DLBCL and 5 with FL. As of 9 April 2021, in the tolerated regimen at dose levels above 0.4 mg, there is an ORR of 40% for CC-99282 monotherapy, with 3 CR (8.5%) and 7 PR (20%). Neutropenia (54%), thrombocytopenia (9%) and febrile neutropenia (6%) are the commonest grade 3/4 TEAEs [95]. Furthermore, the synergistic effect of CC-99282 with anti-CD20 monoclonal antibody enhanced the cell-killing effect of macrophages and human natural killer cells mediated phagocytosis, antibody-dependent cytotoxicity, and antibody independent phagocytosis when the two drugs are used in combination [144]. The latest research results confirm that R-CHOP and CC-99282 can achieve excellent synergistic efficacy in the treatment of untreated aggressive B-cell lymphoma, and CC-99282 has shown manageable safety [143].

#### KT-333

The expression of genes involved in the growth, survival, differentiation, stemness and cell–cell interactions of cells is regulated by STAT3. When cytokines and growth factors are activating STAT3, it leads to tumor growth and hinders anti-tumor immunity. Thus, STAT3 has an essential function in growth factor and cytokine signaling in both normal and malignant cells. Phosphorylated STAT3 levels have been found to be elevated in many human hematologic malignancies, and STAT3 has historically been considered a “undruggable” protein [146, 147]. As a degrader based on the TPD techniques, KT-333 has a high degree of selectivity and ability to target STAT3. Durable tumor regression was observed in preclinical studies in patients with STAT3-dependent T-cell lymphomas following once-weekly intravenous injection of KT-333. STAT3 was efficiently and selectively degraded by KT-333 in four anaplastic T-cell lymphoma cell lines [146, 147].

As of July 10, 2023, in a clinical trial involving 21 patients, these patients received treatment at five different dose levels. The disease types of the participants included 13 solid tumors (ST), 3 cutaneous T-cell lymphomas (CTCL), HL, B-cell NHL, large granular lymphocyte leukemia (LGLL), and peripheral T-cell lymphoma (PTCL), with 1 case of each disease. There have been no reports of severe adverse reactions or dose-limiting toxicities related to KT-333. Grade 1/2 adverse reactions are most common, including symptoms such as anemia, nausea, fatigue, and constipation. For evaluable patients, 1 CTCL patient showed a PR to the disease, while 3 ST patients showed a stable disease (SD) response. In addition, blood data showed a strong, dose-dependent, sustained degradation trend of STAT3 in PBMC. More importantly, KT-333 also led to a dose-dependent downregulation of plasma inflammation biomarkers regulated by STAT3, such as serum amyloid A and C-reactive protein [91].

#### KT-413

DLBCL with myeloid differentiation primary response 88 (MYD88) oncogenic mutations was found to have poor survival after first-line treatment. MYD88 gene mutations were found to cause activation of the NF-κB pathway, which was implicated in the upregulation of pro-inflammatory cytokines as well as genes involved in the survival and proliferation of tumor cells. A key component of the Myddosome complex, IRAK4, is required for NF-kB pathway activation. As another effective and selected heterobifunctional small molecule degrader, KT-413 has been shown to target IRAK4 and IKZF1/3. In the MYD88-mutant preclinical model of DLBCL, KT-413 was shown to have activity in inducing partial or complete tumor regression. In the MYD88-mutant patient-derived xenograft model, KT-413 also showed strong tumor growth inhibition [148]. A clinical study in patients with relapsed or refractory B-cell NHL who have received KT-413 is ongoing (NCT05233033) and no data have been published.

#### NX-5948

NX-5948 is another heterobifunctional small molecule degrader targeting BTK with therapeutic efficacy in lymphoma patients. NX-5948 selectively degrades BTK in hematologic and brain malignancies and avoids degradation of IKZF1/3. Preclinical data showed that NX-5948 degraded 50% of cellular BTK in PBMCs and lymphoma cell lines. Degradation of mutant BTK-C481S in cells can also be induced by NX-5948. In animal studies, BTK degradation in cells was clearly observed after administration of NX-5948. The capability for penetrating the central nervous system (CNS) is one of the more surprising features of NX-5948, which makes it active against malignant tumors in the brain. BTK in intracranial cells can be degraded by NX-5948, which facilitates the reduction of intracranial tumor load. The levels of NX-5948 detected in CSF were the same as those of unbound NX-5948 in plasma after administration of oral NX-5948 to mouse. The potent BTK degrading activity of NX-5948 and its ability to penetrate the CNS gives NX-5948 great potential towards the therapy of B-cell malignancies and even CNS lymphomas [149].

As of June 9, 2023, 14 patients participated in the clinical trial of NX-5948 oral treatment (NCT05131022). The disease types of these participants include 1 case of FL, 2 cases of marginal zone lymphoma (MZL), 3 cases of MCL, 4 cases of DLBCL, and 4 cases of CLL. To date, there have been no reports of severe adverse reactions or dose-limiting toxicities associated with NX-5948. The most common TEAEs were petechiae/bruising (57.1%), thrombocytopenia (35.7%), and nausea (35.7%). NX-5948 exhibits dose-proportional pharmacokinetics, regardless of the dose of NX-5948 received, the type of tumor, or the initial level of BTK, all patients observed robust, rapid, and sustained BTK degradation. In the evaluable responses of 3 CLL patients, 1 case showed PR and 2 cases had SD [92].

### Multiple myeloma

Multiple myeloma (MM) is a malignant tumor caused by a monoclonal expansion of plasma cells in the bone marrow and is currently incurable [150]. Since immunomodulatory drugs (like thalidomide, pomalidomide) and proteasome inhibitors (like bortezomib) were used to treat patients with MM as standard therapy, the MM patient’s median survival has been extended from 3–4 years to approximately 7–8 years, with significant improvement in outcomes [151, 152]. Although patients with MM show good response to initial chemotherapy, the endgame result is always relapse and resistance to treatment [153]. In recent years, the use of TPD to treat relapsed or refractory MM patients has become a therapeutic option. These drugs (like CC-92480, CC-220, CFT-7455, ICP-490) are able to inhibit tumor growth and proliferation effectively by degrading the pathogenic proteins associated with tumorigenesis.

#### CC-92480 (Mezigdomide)

CC-92480 acts as a CELMoD with potent tumor killing and immunostimulatory effects similar to or even exceeding those of IMiD, thus CC-92480 maximally induces degradation of IKZF1/3 [96, 154]. It was also shown that deficiency of IKZF1/3 induces apoptosis and inhibits the proliferation of MM cells and other B-cell derived malignancies [84, 86, 155]. Multiple clinical trials of CC-92480 alone or in combination to treat hematologic malignancies are currently underway. CC-92480 combined with bortezomib (BORT) and dexamethasone (DEX) for the treatment of relapsed or refractory MM phase I/II clinical trial has enrolled 19 patients (NCT03989414). In the observed period, the ORR for all doses reached 73.7%, including 3 patients with stringent complete response (sCR) (15.8%) and 1 patient with CR (5%). TEAEs of grade 3/4 were observed in 18 patients (94.7%) [156]. In 2022, another phase I/II clinical trial (NCT03374085) to treat relapsed or refractory MM with CC-92480 in combination with DEX enrolled 101 patients. The ORR was 39.6%, 2 patients with sCR (2.0%), 3 patients with CR (3.0%), 18 patients with very good partial response (VGPR) (17.8%), and 17 patients with PR (16.8%). In this study, a considerable 89.1% of the 90 patients experienced grade 3/4 TEAEs. The most prevalent hematologic TEAEs of this grading included neutropenia, affecting 74.3% of patients, followed by anemia and thrombocytopenia, which impacted 32.7% and 25.7% of patients, respectively [96]. In addition, CC-92480 has also been shown to have strong synergistic effects with DEX, BORT and anti-CD38 monoclonal antibodies [157]. According to the latest reports, mezigdomide (MEZI) has been confirmed to be used in combination with elotuzumab (ELO), daratumumab (DARA), and DEX to treat relapsed or refractory MM. In this study, the preliminary efficacy and safety of the MeziEd regimen composed of MEZI, ELO, and DEX, as well as the MeziDd regimen composed of DARA and DEX, were evaluated in patients with relapsed or refractory MM. As of May 25, 2023, 57 patients participated in the MeziDd regimen. In the evaluable safety population (*n* = 56), the ORR was 75.0%, including 2 cases of sCR (3.6%), 8 cases of CR (14.3%), 16 cases of VGPR (28.6%), and 16 cases of PR (28.6%). In this population, 43/56 (76.8%) of patients experienced grade 3/4 TEAEs, including 30 patients (53.6%) with neutropenia, 11 patients (19.6%) with infections, and 4 patients (7.1%) with thrombocytopenia. On the other hand, the MeziEd regimen had 20 patients participating. Among all patients who had received anti-CD38 monoclonal antibody therapy (100%), the ORR of the MeziEd regimen was as high as 56% [158].

#### CC-220 (Iberdomide)

CC-220, also known as a CELMoD, demonstrates approximately 20 times greater affinity for CRBN compared to IMiDs. This superior binding capacity enables more effective degradation of IKZF1/3 [159]. CC-220 exerts myeloma cell killing and anti-proliferative effects after degradation of IKZF1/3, and also stimulates antimyeloma immunoreactivity of T cells and NK cells [160, 161]. The combination of CC-220 and DEX in the treatment of relapsed or refractory MM demonstrated manageable safety and an ORR of 31.9%. In another study of 38 patients with relapsed or refractory MM treated with a combination of CC-220 and DEX, these patients had previously received treatment targeting B-cell maturation antigen (BCMA), CD38 monoclonal antibodies, proteasome inhibitors, lenalidomide, or pomalidomide (NCT04975997). As of April 15, 2022, an ORR of 36.8% was recorded, comprising of 2 instances of CR (5.3%), 7 instances of PR (18.4%), and 5 instances of VGPR (13.2%). There were 30 patients (78.9%) with grade 3/4 TEAEs, the commonest being neutropenia (50%), anemia (28.9%), leucopenia (23.7%) and thrombocytopenia (21.1%) [97].

In other B cell derived malignancies (like DLBCL), CC-220 was also found to mediate the degradation of IKZF1/3. The clinical trial of CC-220 monotherapy or combined with the anti-CD20 monoclonal antibody (NCT04464798) enrolled 46 patients (including 18 DLBCL and 10 FL). Among the 38 patients who showed observable responses, the ORR was 55%, CR was 32%. The main TEAEs of grade3/4 were thrombocytopenia (13%), anemia (15%), and neutropenia (49%). Preliminary analyses also suggested a dose–response correlation for CC-220. The evidence indicated a trend towards enhanced IKZF1/3 degradation with increasing concentrations of the drug [162].

#### CFT-7455

CFT-7455 is a novel optimized CELMoD with 800–1600 times higher affinity for CRBN compared to pomalidomide, allowing for the rapid degradation of IKZF1/3. Another advantage of CFT-7455 is its ability to maintain activity in models of resistance or insensitivity to IMiDs [163]. Comparison of CFT7455 and CC-92480 concentrations after administration for 48 h in plasma and tumor showed significantly higher concentrations of CFT7455 than CC-92480. This suggests that CFT7455 can mediate the sustained degradation of IKZF1/3 in a preclinical model with a longer exposure time [164]. In 5 patients receiving monotherapy with CFT7455 (NCT04756726), early pharmacodynamic data showed sustained profound degradation of IKZF3 (approximately 100%), reduction in serum free light chains (up to 72%), and neutropenia in 3 patients. Disease stability was observed in an additional 34 patients, suggesting a response to treatment with CFT7455 in MM patients [164]. The clinical trial is currently ongoing and the final results of CFT7455 are yet to be announced.

#### ICP-490

MM patients treated with IMiD for an extended duration of time can develop resistance to downregulation of CRBN, which ultimately leads to tumor recurrence. In addition, IMiD produces teratogenic side effects through CRBN-mediated degradation of ΔNp63/TAp63, PLZF1 and SALL4. ICP-490 selectively, potently and deeply degrades IKZF1/3 at an IC50 below nanomolar, but does not significantly degrade GSPT1, PLZF1, SALL4, IKZF2 and IKZF4. Its therapeutic efficacy is superior to that of currently approved IMiDs in various MM and NHL tumor models. What’s more, ICP-490 is not cytotoxic to normal human cells (such as PBMC and HEK293 cells) [165]. The effectiveness and safety of ICP-490 in patients with relapsed or refractory MM is undergoing clinical trials (NCT05719701).

## The novel opportunities in TPD

**Fig. 5:**
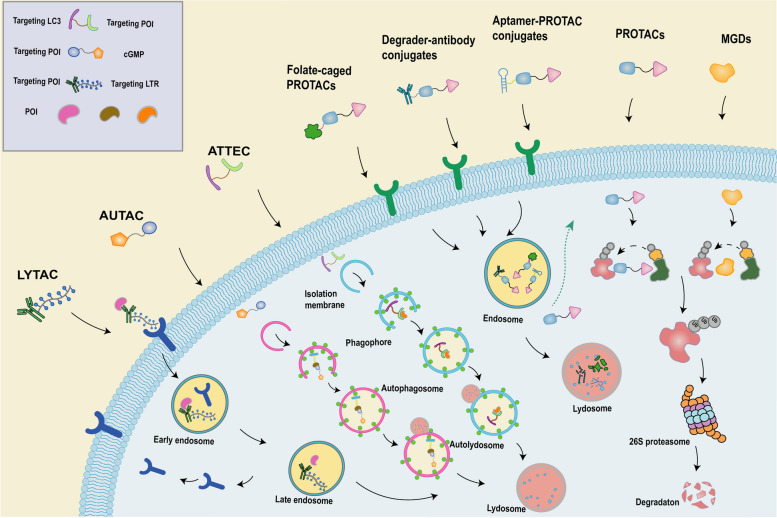
Schematic diagram of the mechanism of action of various TPD-based techniques. PROTACs and MGD bind to POI, and subsequently mediate the ubiquitination of POI. The polyubiquitinated POI was transported to the 26S proteasome for degradation. Degradant-antibody conjugates, aptamer-PROTACs conjugates, folate-caged PROTACs bind specifically to receptors on the surface of target tissues (like tumor cells), leading to the internalization of pro-PROTACs. The internationalized pro-PROTACs are subsequently transferred to the lysosomal compartment of the cell, where the pro-PROTACs complex are dissociated by protein hydrolysis and an acidic environment, and the PROTACs are released into the cytoplasm as a payload to perform their function. Autophagosome-tethering compounds (ATTECs) bind POI and LC3, binding induces autophagosome formation and subsequent fusion of autophagosomes with lysosomes leading to POI degradation. Autophagy-targeting chimeras (AUTACs) consist of a cGMP-based degradation tag, a linker, and a POI-targeting warhead. AUTACs also induce autophagosome formation and subsequent fusion of autophagosomes with lysosomes leading to POI degradation. Lysosome-targeting chimeras (LYTACs) consist of antibodies or small molecules coupled to ligands that bind lysosome-targeted receptors (LTRs) such as ASGPR and CI-MPR. ASGPR is expressed only in the liver, whereas CI-MPR is expressed ubiquitously in all tissues of the body. ASGPR or CI-MPR is endocytosed along with the LYTACs and the POI, and subsequently the POI is degraded by the lysosome, and ASGPR or CI-MPR was recycled to the plasma membrane for recycling

Numerous proteins targeting degraders, currently undergoing clinical trials, have demonstrated effective results in treating patients with hematologic malignancies. Concurrently, the constant innovation in TPD has prompted a surge in novel therapeutic approaches. Various optimized, improved PROTACs technologies are designed to improve the form, range, specificity and security of TPD targeted POI delivery. Prodrug-based PROTACs (Pro-PROTACs) are proficient in enabling accurate, efficient, and minimally toxic degradation of POI due to their inherent ability to locally release active PROTACs within the tumor site. Complementary techniques rooted in the lysosomal pathway can rectify the shortcomings of UPS in protein degradation scenarios. In the following sections, we will delve into contemporary advances on the frontier of TPD **(**Fig. 5**)**.

### TPD technology based on proteasomal degradation pathway

#### Prodrug-based PROTACs involving receptor-mediated internalization

Degrader-antibody conjugates(DACs) represent the new entities that bind PROTACs to monoclonal antibodies through specific chemical linkers, and belong to the receptor-mediated internalization prodrug-based PROTACs (pro-PROTACs) [166]. As an embryonic concept in the field of TPD, the design of DACs is inspired by antibody–drug conjugates (ADCs), where DACs can target cancer-associated cells through their unique and efficient mechanism of action [167, 168]. Firstly, the antibody portion of the DACs specifically combines with antigens expressed on target tissue surface (like tumor cells), resulting in the internalization of the DACs complex. The internalized DAC is then transported to the lysosomal compartment of the cell, where the DAC complex linker is dissociated by the acidic environment and proteolytic hydrolysis, and the PROTACs, which acts as a payload, is released into the cytoplasm to function [168, 169]. DACs offer the following advantages over simple PROTACs technology: 1)targeting PROTACs molecules to specific tissues or tumors through antigen recognition by DACs; 2)delivering PROTACs with inferior capacity for cell permeation, water solubility, and low oral bioavailability in vivo [168]. DACs are at an embryonic stage, and although various such entities have been created, most of them have primarily used BRD4 targeted payloads and thus have limited types of PROTACs that can be connected, with few applications in the hematologic malignancy domain.

Folate-caged PROTACs, a novel type of pro-PROTACs, created by conjugating a VHL E3 ubiquitin ligase with a folate molecule [170]. Folate receptor α was shown overexpression in selected tumors of several types. Therefore, PROTACs can exert a powerful therapeutic effect when specifically delivered to these cells [171]. Several folate-caged PROTACs have been used to target degradation of ALK, BRD, and MEK [170]. In addition, the first CRBN based folate-caged PROTACs was successfully developed via a reductively cleavable disulfide-containing linker coupling folic acid to the pomalidomide-derived ALK degrader (MS4048) [172]. Folate-caged PROTACs can mitigate the therapeutic toxicity associated with PROTACs and broaden the therapeutic window for TPD.

Aptamer-PROTACs conjugates (APCs) are another receptor-mediated internalization pro-PROTACs, when aptamer (a single-stranded oligonucleotide sequence) is coupled to PROTACs, with the advantage of low immunogenicity and high tissue penetration [166, 173]. The first APCs were successfully developed after BET targeted PROTACs with the currently clinically evaluated most advanced aptamer AS1411 [174–176]. Compared with PROTACs monotherapy, APCs have enhanced BET degradation and anti-tumor efficacy, as well as reduced toxicity [175]. However, the APCs are currently used to specifically target breast cancer, and we anticipate the early application of this technology to hematologic malignancies.

#### Pro-PROTACs activated by light and other mechanisms

Light is an external factor that allows for the precise delivery of drug candidates through photodynamic therapy, so modulating the activity of PROTACs through light has become a highly valuable therapeutic modality. The currently available pro-PROTACs based on photoactivation can be mainly categorized into photocaged PROTACs and photoswitchable PROTACs. Photocaged PROTACs under the circumstances without light, the photocleavable caging group binds to the parent PROTACs and inhibits its binding to E3 ligase or POI. Activated PROTACs was delivered to specific tissues under the conditions of light to perform its function. Photoswitchable PROTACs utilize the azo-containing linkers to connect the E3 ligand to the POI warhead instead of a conventional alkyl or polyether linker. Under specific wavelengths of light, “cis” and “trans” photoswitchable PROTACs can be interconverted to achieve different biological activities [166]. The use of light to modulate the activity of PROTACs can control the efficacy under special conditions, and this emerging design approach has great prospect in hematologic malignancies.

Pro-PROTACs activated by other mechanisms were developed to reduce the tumor off-target toxicity of conventional PROTACs molecules, such as nitroreductase-responsive PROTACs, radiotherapy-triggered PROTACs, reactive oxygen species-responsive PROTACs. Utilizing different conditions of action such as nitroreductase, reactive oxygen species, and radiotherapy, pro-PROTACs enable targeted delivery of potent degraders to tumor cells, thus decreasing the exposure of healthy organs to the drug and increasing the safety of treatment with conventional PROTACs [166].

#### Emerging PROTACs technologies of other types

Trivalent PROTACs are a modified TPD technology that promotes POI degradation by increasing cooperativity and affinity. Compared with conventional PROTACs, trivalent PROTACs exhibit sustained and superior degradation efficacy and enhanced anticancer activity [177]. Bio-PROTACs are engineered E3 ligases in which the substrate recognition domain has been designed for expression of a protein structure or target peptide [178]. Bio-PROTACs’s warheads are based on nanobody, DARPins or protein-binding peptides, thus retaining higher specificity, lower off-target effects and shorter discovery phases. Moreover, Bio-PROTACs contain E3 ligase inherently, so it can be expressed naturally independent of endogenous E3 ligase [179].

### TPD technology based on lysosomal pathway

Approximately 40% of genetically encoded protein products are extracellular and membrane-associated proteins that are implicated in cancer, aging and autoimmune diseases. However, these targets are currently not degradable by PROTACs and molecular glue using UPS, so selective degradation of these proteins is an urgently needed new strategy [7, 180]. LYTACs, AUTACs, and ATTECs degrade “undruggable” targets via the lysosomal pathway. These emergent protein degradation tactics extend the range of POI to membrane proteins, extracellular proteins, and organelles. For instance, LYTACs can degrade extracellular proteins [19], ATTECs can degrade lipid droplets [181], and AUTACs can mediate mitochondrial degradation [182]. While there are no drugs based on the LYTACs, AUTACs and ATTECs technologies in clinical trials, their specific targets of action are appealing to treat the hematologic malignancies.

## The current dilemma of TPD

Despite the numerous advantages of TPD, there are still inherent drawbacks requiring significant amelioration. The initial discussion focused on pharmacokinetics and design accessibility. Most targeted protein degraders have more rotatable bonds, high lipophilicity and high molecular weight, thus are deficient in cell permeability, aqueous solubility and oral bioavailability [183–185]. The com plicated chemical space of PROTACs resulting in its restriction of pharmacokinetics/pharmacodynamic, which makes it undesirable to administer orally [186, 187]. In addition, the event-driven pharmacological of TPD lead to complete degradation of POI, which will require a long recovery period to re-establish the intracellular protein degradation pool. Therefore, this may result in unforeseen drug toxicity risks [188].

E3 ligase, is another related challenge. Firstly, the human genome is reported to encode over 600 E3 ubiquitin ligases, however just one handful of these, such as MDM2, IAPs, CRBN and VHL, were already used in TPD technology [50]. Secondly, E3 ligase acts as a critical point on the TPD pathway, when the gene for E3 ligase is altered or its expression is reduced, resistance to the targeted protein degraders can develop. CRBN is an important binding protein for TPD, and it has been found that the frequency of aberrations in CRBN elevates with increased exposure to IMiD [189]. The efficacy and safety of TPD will be further enhanced by optimizing the structure of PROTACs, rationally designing the molecular glue, and exploring more E3 ligases.

## Conclusion

The application of TPD techniques in hematologic malignancies has provided a novel perspective on protein targeted therapies over two decades of advancement. As two of the most well-developed techniques for TPD, various drugs based on PROTACs and molecular glue have shown substantial efficacy against hematologic malignancies. Drugs such as CC-122, CC-99282, CC-92480, NX-2127, NX-5948 not only have a large number of ongoing clinical trials, but also show positive results in published data. The TPD technique, as a strategy for the precise degradation of proteins in tumor cells, holds promise for the precise treatment of hematologic malignancies. Various novel PROTACs techniques with improvements in delivery format, specificity and safety can remedy the shortcomings found in PROTACs in the past. Other TPD techniques including LYTACs, AUTACs, ATTECs, CMA-based degraders, double-mechanism degraders, and chaperone-mediated protein degraders will improve the range of targeted proteins and degradation efficiencies. There is no doubt that TPD techniques will not only become a valuable accessory for biomedical research, it will also become an important component of future targeted drug development.

## Data Availability

Not applicable.

## Abbreviations

ALL: Acute lymphoblastic leukemia
AML: Acute myeloid leukemia
APCs: Aptamer-PROTACs conjugates
ATTECs: Autophagosome-tethering compounds
AUTACs: Autophagy-targeting chimeras
BNIP3L: BCL2-interacting protein 3 like
BTK: Bruton’s tyrosine kinase
CRBN: Cereblon
CELMoDs: Cereblon E3 ligase-modulating drugs
CLL: Chronic lymphocytic leukemia
CR: Complete remission
CRi: Morphologic complete remission with incomplete blood count recovery
CTCL: Cutaneous T-cell lymphomas
DCAF15: DDB1 and Cul4-associated factor 15
DACs: Degrader-antibody conjugates
DLBCL: Diffuse large B cell lymphoma
FL: Follicular lymphoma
GSPT1: G1 to S phase transition 1
HSCT: Hematopoietic stem cell transplantation
IAPs: Inhibitor of apoptosis proteins
IMiDs: Immunomodulatory drugs
IKZF1: Ikaros
IKZF3: Aiolos
LGLL: Large granular lymphocyte leukemia
LYTACs: Lysosome-targeting chimeras
MCL: Mantle cell lymphoma
MDM2: Mouse double minute 2 homolog protein
MDS: Myelodysplastic syndrome
MetAp-2: Methionine aminopeptidase-2
MGDs: Molecular glue degraders
MLFS: Morphologic leukemia-free state
MZL: Marginal zone lymphoma
MM: Multiple myeloma
NHL: Non-Hodgkin’s lymphoma
ORR: Overall response rate
PBMCs: Peripheral blood mononuclear cells
PD: Pharmacodynamics
PK: Pharmacokinetics
PROTACs: Proteolysis-targeting chimeras
Pro-PROTACs: Prodrug-based PROTACs
POI: Proteins of interest
PTCL: Peripheral T-cell lymphoma
sCR: Stringent complete response
SD: Stable disease
STAT3: Signal transduction and activation of transcription 3
ST: Solid tumor
TEAEs: Treatment-emergent adverse events
TPD: Targeted protein degradation
UPS: Ubiquitin proteasome system
VGPR: Very good partial response
VHL: Von Hippel-Lindau

## Data availability

No datasets were generated or analysed during the current study.
